# Editorial: Prognostic factors in hepatocellular carcinoma

**DOI:** 10.3389/fmed.2023.1331207

**Published:** 2023-11-28

**Authors:** Liliana Chemello, Pradeep Kumar Shukla, Marcello Dallio

**Affiliations:** ^1^Department of Medicine-DIMED, University of Padova, Padua, Italy; ^2^Department of Pathology, St. Jude Children's Research Hospital, Memphis, TN, United States; ^3^Department of Gastroenterology, Hepatology and Digestive Endoscopy, University of Campania Luigi Vanvitelli, Naples, Italy

**Keywords:** HCC, tumor prognosis, hepatocellular carcinoma, immunotherapy, biomarkers

Hepatocellular carcinoma (HCC) represents the third leading cause of cancer-related mortality worldwide (1). Its occurrence is geographically linked to the prevalence of hepatitis B virus (HBV) infection, with incidence rates of 80% in Asia and Africa, while other etiologies (i.e., chronic hepatitis C and alcohol or metabolic-related) are causing an increase in HCC, particularly in developed countries (2). The survival rate ranges from several years to a few months, mainly in accordance with the disease stage and progression threshold of the tumor, and it is often associated with chronic liver disease and cirrhosis, at least in Europe and North America.

The choice for the best treatment is guided by the prognostic classification of the Barcelona Clinic Liver Cancer (BCLC) (3), which takes into consideration tumor burden, liver function, particularly with the presence of cholestasis or portal hypertension signs, and performance status. Thus, it also suggests a series of interventions by loco-regional and combined strategies involving focal (i.e., surgical resection or ablation) or systemic treatments, but the only curative chance is liver transplant.

Early diagnosis using new biomarkers, combined with artificial intelligence programs (4), could extend survival expectations by a simplified approach to patients with HCC. Therefore, the identification of serum glycobiomarkers associated with HCC using Lectin microarrays represents a promising step toward the early detection of cases with HCC. Zhang Y. et al. analyzed the specific glycosylation pattern of HCC proteins, identifying a unique glycan signature associated with HCC. This study showcases the potential of innovative techniques in the field of biomarker discovery (i.e., glycomic and glycoproteomic technologies) and highlights the importance of further validation and clinical translation for the early detection of HCC and improved management of patients.

Moving on to the prognosis of HCC, the meta-analysis proposed by Zhang X. et al. involving 10,369 patients with HCC, evaluated the association between different Aspartate Aminotransferase-to-Platelet Ratio Index (APRI) levels and overall survival (OS). Patients with high APRI (i.e., ≥1) had an increased risk of death by 77% compared to cases with low levels. The choice of APRI as a biomarker is noteworthy because it is a simple and cost-effective tool that relies on routine blood tests to estimate the severity of liver fibrosis and inflammation and has now been added as an independent prognostic factor and effective early screening method in cases with HCC. However, it is essential to recognize that this study is one piece of the puzzle, and further research and clinical validations are required to establish APRI as a reliable and practical tool in the context of HCC.

Moreover, Liu R. et al. have introduced the prognostic role of the neurotrophin factor-3 (NTF3), a protein belonging to the growth factor family, which is associated with the progression of multiple cancers. They focused on its potential influence on tumor immunity. By performing a bioinformatic analysis of the different expressions of NTF3 mRNA in liver cancer and normal tissues, they found that NTF3 is under-expressed in HCC and it could be correlated to a poor tumor prognosis. These results highlight that NTF3 could be a potential prognostic biomarker for HCC, but further studies considering the entire tumor environment's involvement are needed.

Staying on the subject, Mao et al. have also focused on promising prognostic markers in HCC. They studied the role of the ribonucleotide reductase M2 subunit (RRM-2), a small subunit of the ribonucleotide reductase complex. They proved that RRM-2 is up-regulated in HCC, and it is positively related to its poor prognosis. The most interesting result was the relationship between the high expression of RRM-2 in HCC and PD-1, PD-L1, and CTLA-4, the immune checkpoints responsible for tumor immune escape. Therefore, the tumor immune escape may be influenced by RRM2 pathways, and thus RRM2 may be proposed as a therapeutic target of immunotherapy (IT) in HCC.

The diffusion of new radiation techniques (i.e., 3D, stereotactic, and proton beam therapy), and from 2017, the arrival of the HCC “IT era” (i.e., *PD-1, VEGF, CTLA-4 inhibitors*) benefited more patients with end-stage HCC by significantly prolonging life-expectancy.

Kaewdech et al. in a retrospective cohort study of 317 treatment-naïve cases with unresectable HCC staged BCLC A or B performed an analysis to identify cases with a poor OS after trans-arterial chemoembolization (TACE) as the first line. Forty-two cases were deceased within 6 months after the first TACE session, showing a significantly higher FAIL-T score, a model based on AST, ALT, AFP, and tumor size and number. Cases stratified with FAIL-T score <8 or ≥8 statistically differed in OS with 23.8 *vs*. 4.6 months, respectively. This study presents a prognostic model for cases treated with TACE at the intermediate HCC stage and facilitates the choice to move directly to systemic treatment in cases with FAIL-T score ≥ 8, which may be associated with the poor effectiveness of TACE.

The purpose of evaluating the effects of chemotherapy (CHT) and radiotherapy (RT) on the prognosis of unresectable HCC cases with a portal or hepatic vein invasion has been addressed in the paper of Qiu et al. using the SEER registry database including 2,614 cases and balanced by propensity score matching with an untreated group. Treated cases had longer survival, particularly in cases that received a combined protocol of CHT and RT (10 *vs*. 5 months, *p* < *0.001*). In this patients' subset, the tumor stage (size, number, or metastatic diffusion) and AFP level were identified as independent risk factors of OS. Macrovascular invasion has been recognized as the one important prognostic factor affecting OS in cases with HCC, as these cases have already lost the chance of receiving radical surgery at the diagnosis time.

Since further studies on HCC therapeutic strategies are needed in advanced HCC, the evaluation of a new strategy by the combination of locoregional treatment with VEGF-α inhibitor therapy is certainly innovative. Specifically, Liu S. et al. have retrospectively evaluated the OS in 76 patients with BCLC stage C HCC, who had undergone TACE alone or in combination with Apatinib, in light of the limited efficacy of Sorafenib in the Asia-Pacific region. They showed that the median survival time in the TACE-Apatinib *vs*. TACE-alone group was 10 *vs*. 6.2 months, respectively. Although for the small number of patients, this result could open the way for prospective evaluations on larger cohorts and from more heterogeneous geographical areas.

Furthermore, novel clinical concepts on the evaluation and definition of treatment response to HCC by the application of agreed criteria can amplify the patient's benefit. Lee et al. give us a note of caution during the evaluation of response to therapy in solid advanced tumors, such as HCC. Pseudoprogression (PP) of the tumor described by the immune response evaluation criteria in solid tumor (iRECIST) may not be an actual progression of HCC but only a radiological increase of the tumor size by infiltration of immune cells or cytotoxic T cells that induce edema and necrosis phenomena in the tumor. In this study, 158 cases with HCC received Nivolumab after Sorafenib failure, showing a low rate of PP pattern which may indicate the futile benefit of continuing IT and the need for a therapeutic strategy change.

In this Research Topic, we discussed the recent advances for early diagnosis of HCC, that is essential for applying a curative therapy in patients, as the research on the specific glycosylation pattern of HCC proteins. We also focused on promising biomarkers, introduced as prognostic factors in cases with advanced HCC disease, like: the liver and tumor tissues expression of NTE-3 or RRM-2 related to poor tumor prognosis, and the choice to apply APRI, as biomarker independently associated to OS of patients with HCC.

These strategies seem to offer some potentially advantages to expand patients' survival even if, the response rate target also needs some major improvements to support the burden of the disease, especially by introduction of new or combined CHT-RT-IT protocols, particularly targeted to the advanced stage of HCC disease.

## Author contributions

LC: Conceptualization, Validation, Writing – original draft, Writing – review & editing. PS: Conceptualization, Validation, Writing – original draft, Writing – review & editing. MD: Conceptualization, Validation, Writing – original draft, Writing – review & editing.
